# Detection of myocardial infarction by postmortem CT combined with radiomics-based machine-learning analysis

**DOI:** 10.1186/s41747-026-00800-4

**Published:** 2026-09-11

**Authors:** Nilay Chandra Barman, Aditya Rastogi, Alexander Radbruch, Julian Alexander Luetkens, Daniel Wittschieber, Maria Hahnemann

**Affiliations:** 1 Institute of Forensic Medicine, University Hospital Bonn, University of Bonn, Bonn, Germany; 2 Department of Neuroradiology, University Hospital Bonn, University of Bonn, Bonn, Germany; 3 Division for Computational Radiology & Clinical AI, Clinic for Neuroradiology, University Hospital Bonn, Bonn, Germany; 4 Department of Diagnostic and Interventional Radiology, University Hospital Bonn, University of Bonn, Bonn, Germany

**Keywords:** Forensic imaging, Machine learning, Myocardial infarction, Postmortem imaging, Radiomics

## Abstract

**Background:**

To determine whether quantitative radiomic features extracted from noncontrast postmortem computed tomography (PMCT) can reliably detect autopsy-confirmed myocardial infarction (MI), and to evaluate the diagnostic performance of machine-learning models for automated MI detection.

**Materials and methods:**

We retrospectively analyzed 106 PMCT examinations (55 MI cases, 51 controls). The left ventricular myocardium was semiautomatically segmented, and radiomic features were extracted. After reproducibility filtering (intraclass correlation coefficient ≥ 0.75), redundancy reduction based on Spearman correlation (ρ > 0.8), and feature selection were performed within a nested stratified cross-validation framework, including no additional selection (N.S.), random forest (RF)-based ranking, and recursive feature elimination with logistic regression (RFE-LR). Seven supervised machine-learning classifiers were trained and evaluated using area under the receiver operating characteristic curve (AUROC) and other metrics.

**Results:**

Logistic regression with N.S. achieved the highest performance, with a mean AUROC of 0.817 ± 0.12 (mean ± standard deviation), with accuracy 0.746 ± 0.05, sensitivity 0.764 ± 0.14, and specificity 0.724 ± 0.13. Support vector machines and RF with N.S. showed comparable performance (AUROC 0.814 ± 0.10 and 0.814 ± 0.08, respectively). Across most classifiers, RF-based and RFE-LR feature selection did not improve diagnostic performance compared with N.S. Four radiomic features were identified by both RF and RFE-LR, being selected in at least two outer cross-validation folds.

**Conclusion:**

Radiomics combined with machine learning demonstrated good performance for MI detection on noncontrast PMCT, emphasizing its potential as an objective, noninvasive tool for cause-of-death assessment, while suggesting a possible translational relevance for clinical contrast-free cardiac imaging.

**Key Points:**

- *Question* Can MI detection on PMCT enable cause-of-death determination in forensic medicine and (clinical) pathology, particularly in settings without autopsy?
- *Findings* Radiomics-based machine learning on noncontrast PMCT showed good diagnostic performance for MI detection (mean AUROC 0.814–0.817).
- *Relevance Statement* Radiomics-based analysis of noncontrast PMCT shows promising potential for the detection of MI without the need for contrast agents or invasive procedures and may support forensic and pathological investigations by enhancing the diagnostic value of routinely acquired PMCT data. However, potential clinical applications require further validation.

**Graphical Abstract:**

**Figure:**
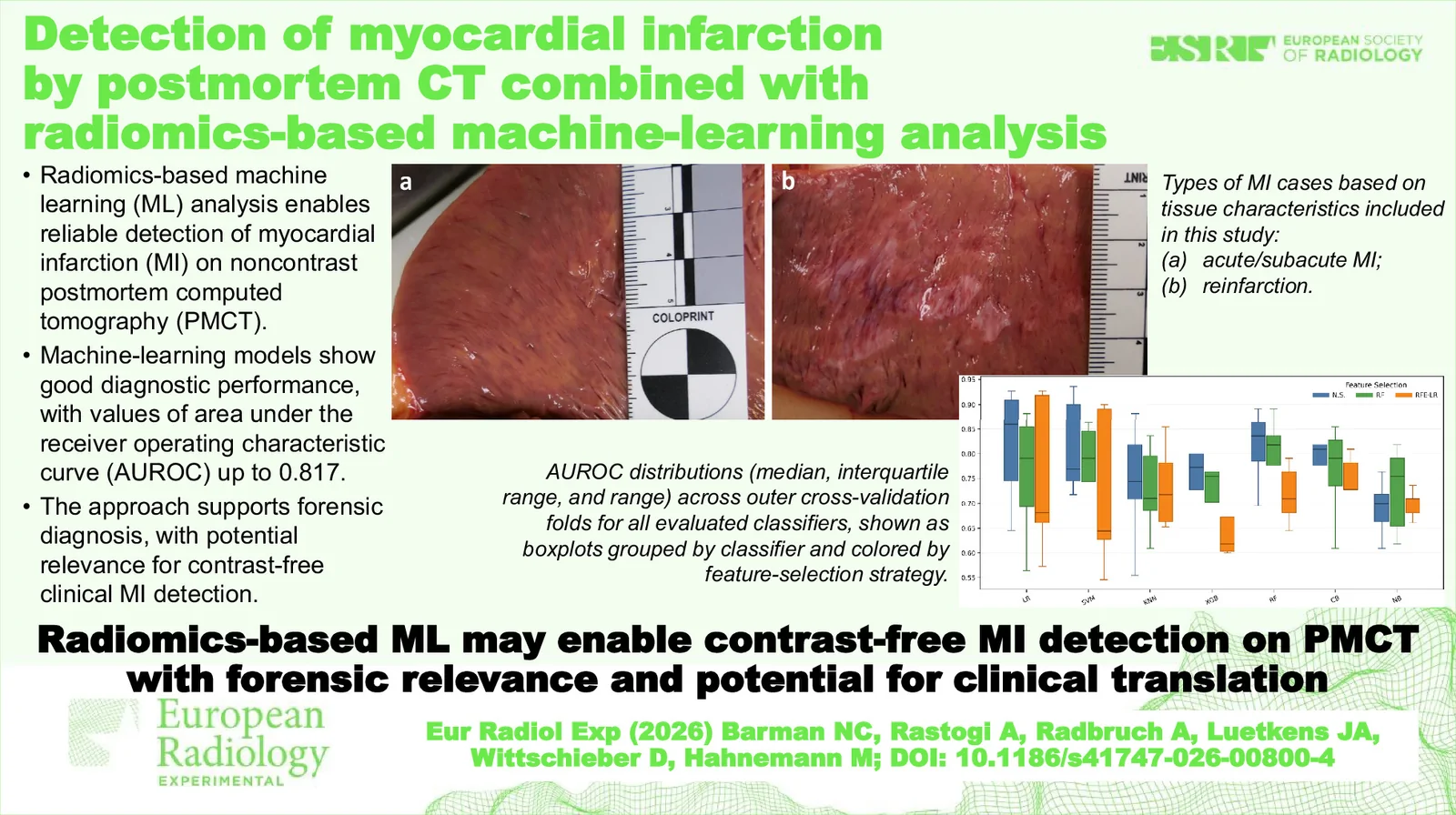

## Background

Myocardial infarction (MI) accounts for approximately one-third of all deaths in individuals over the age of 35 and remains among the leading causes of mortality worldwide [1, 2]. Autopsy continues to be regarded as the gold standard for determining the cause of death in suspected cases of MI.

In recent years, postmortem computed tomography (PMCT) has gained increasing importance and is now primarily used as a complementary method to autopsy, especially in forensic cases. As a non-invasive technique that provides a rapid overview of the entire body, PMCT offers the additional ability to reveal specific and sometimes more detailed information on tissues and organ systems while considering postmortem changes [3]. In cases of fatal cardiac events, PMCT may therefore support a more precise, rapid, and non-invasive identification of MI and their effects on myocardial tissue. This may be especially valuable in situations where conventional autopsy is not possible, *e.g.*, due to a local lack of (forensic) pathological expertise [4, 5], or is declined, such as for religious, cultural, or personal reasons (*e.g.*, in Muslim and Jewish communities) [6, 7], as well as in perinatal death investigations, where non-invasive approaches are often preferred [8]. In such settings, imaging like PMCT may represent the only way to determine or at least narrow down the cause of death. Furthermore, such approaches may not only improve postmortem diagnostics but also enable a more detailed fundamental understanding of MI and its potential risk factors beyond established clinical parameters, as they provide a unique opportunity to directly correlate between imaging findings and pathological outcomes.

A particularly promising approach for analyzing MI in radiological imaging is texture analysis/radiomics. This methodology uses post-processing techniques to evaluate the distribution and relationships of gray-value pixels or voxels within radiological images. Numerous quantitative texture features—often invisible to the human eye—can be extracted from these images [9]. Image texture describes characteristic patterns of gray-value variation [10]. Alterations in tissue structure, such as those caused by MI, may therefore manifest as distinct textural patterns in PMCT. Although infarction-related changes, resulting from ischemia-induced processes such as necrosis, edema, tissue hemorrhages, and fibrosis, are often macroscopically visible at autopsy, partly due to color-related tissue differences, they are not identifiable on noncontrast PMCT. However, these changes may be reflected in subtle intensity and texture variations at the voxel level that can be captured by radiomics, similar to its use in oncology for characterizing tissue heterogeneity and determining tumor phenotypes beyond what is perceptible to the human eye. Supported by artificial intelligence, particularly machine learning, these texture-derived features can be analyzed to identify potential patterns [11, 12].

In clinical settings, texture analysis/radiomics and machine-learning approaches have already demonstrated the ability to detect MI that were not visually discernible in CT and magnetic resonance imaging (MRI) [13–18]. However, the potential of radiomics and machine-learning for the identification of MI in PMCT has not been investigated. Existing PMCT-based attempts largely focus on indirect detection methods, such as the detection of coronary perfusion disorders by means of PMCT angiography [19–21]. However, performing PMCT angiography is very time- and cost-intensive. Developing a reliable postmortem imaging technique for the direct diagnosis of MI without additional angiography would represent a major step forward and offer a valuable, non-invasive tool for cause-of-death determination in forensic medicine and clinical pathology, with comparatively lower time and cost requirements than PMCT angiography. In addition, if translated into clinical settings, novel CT-based methodologies may also prove valuable for the detection of MI in living patients.

Therefore, the present study aims to explore the potential of radiomics and machine-learning techniques applied to PMCT scans of individuals who died from MI as confirmed by forensic autopsy. Specifically, the study evaluates the capability of these methods to differentiate between healthy and infarcted myocardium in PMCT.

## Materials and methods

### Study population

**Fig. 1:**
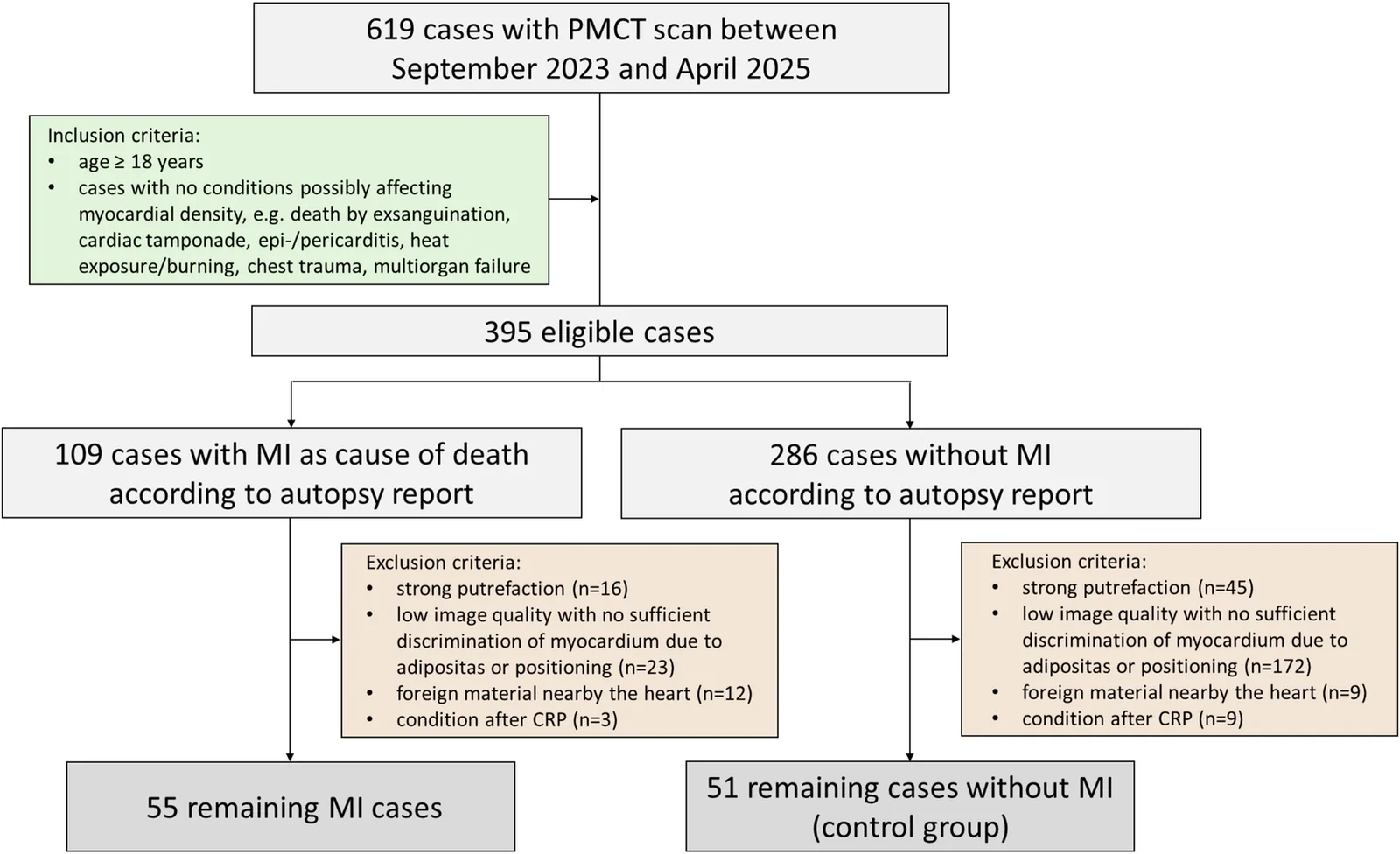
Flowchart indicating inclusion and exclusion criteria in this study. MI Myocardial infarction

Data for this retrospective study were obtained from the institutional database of the Institute of Forensic Medicine, University Hospital Bonn, Bonn, Germany. The study was approved by the local ethics committee (approval number 2025-165-BO). A total of 619 consecutive PMCT examinations were screened, and after applying inclusion and exclusion criteria (Fig. 1), 106 cases were retained for analysis: 55 deceased individuals with MI as the cause of death confirmed by autopsy, and 51 control cases without cardiac pathology.

**Fig. 2:**
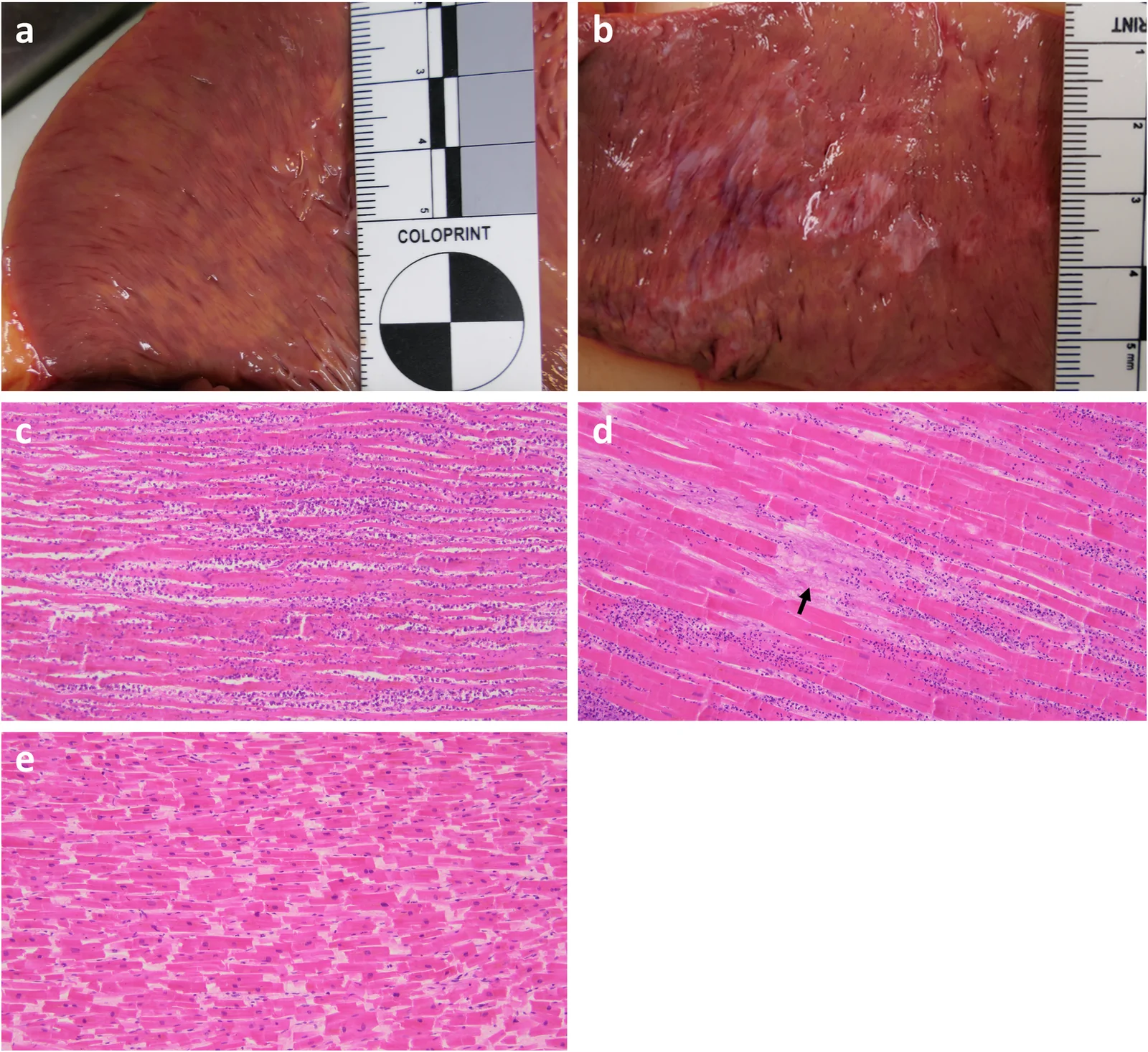
Types of MI cases based on tissue characteristics included in this study: **a** acute/subacute MI, and **b** reinfarction (acute besides old MI); corresponding histological findings (hematoxylin and eosin stain, original magnification 100×) are shown in (**c**, **d**), respectively (black arrow indicating fibrosis). **e** Myocardial histology without evidence of infarction as observed in the control group

The following manifestations of MI were observed: (a) acute/subacute MI—pale myocardial tissue and/or yellowish discoloration (Fig. 2a), and (b) reinfarction (acute besides old MI)—acute pallor adjacent to grayish or whitish scar tissue (Fig. 2b). The corresponding histological findings on H&E staining (original magnification ×100) are shown in Fig. 2c, d for acute/subacute MI and reinfarction, respectively, while Fig. 2e illustrates myocardial histology from a heart without infarction as observed in the control group. In acute/subacute MI (Fig. 2c), myocardial fibers appear hypereosinophilic and wavy with loss of cross-striations and nuclei, accompanied by a prominent neutrophilic infiltrate. In reinfarction (Fig. 2d), features of acute/subacute MI, including hypereosinophilic myocytes and neutrophilic infiltration, are superimposed on pre-existing myocardial damage with focal fibrotic (scar) tissue. These entities of MI were selected for the present study as they are relatively easy and unambiguous to identify macroscopically. Accordingly, MI was primarily determined during forensic autopsy and defined according to macroscopic autopsy findings. In some of the autopsy cases, additional histological examinations of all major organs were routinely performed, allowing MIs in these cases to be histologically verified.

### PMCT image acquisition and image quality

Whole-body PMCT was performed using a 16-slice multi-detector CT scanner (*SOMATOM Emotion 16*; Siemens Healthineers, Erlangen, Germany) with the following parameters: tube voltage 130 kV, tube current 100 mA, slice thickness 1.5 mm, reconstruction increment 1.0 mm, pitch 1.5, field of view 500 mm, and convolution kernel B30s (soft tissue). Images were reconstructed at an in-plane pixel spacing of 0.97 × 0.97 mm² and stored with a matrix size of 512 × 512.

### Image segmentation

**Fig. 3:**
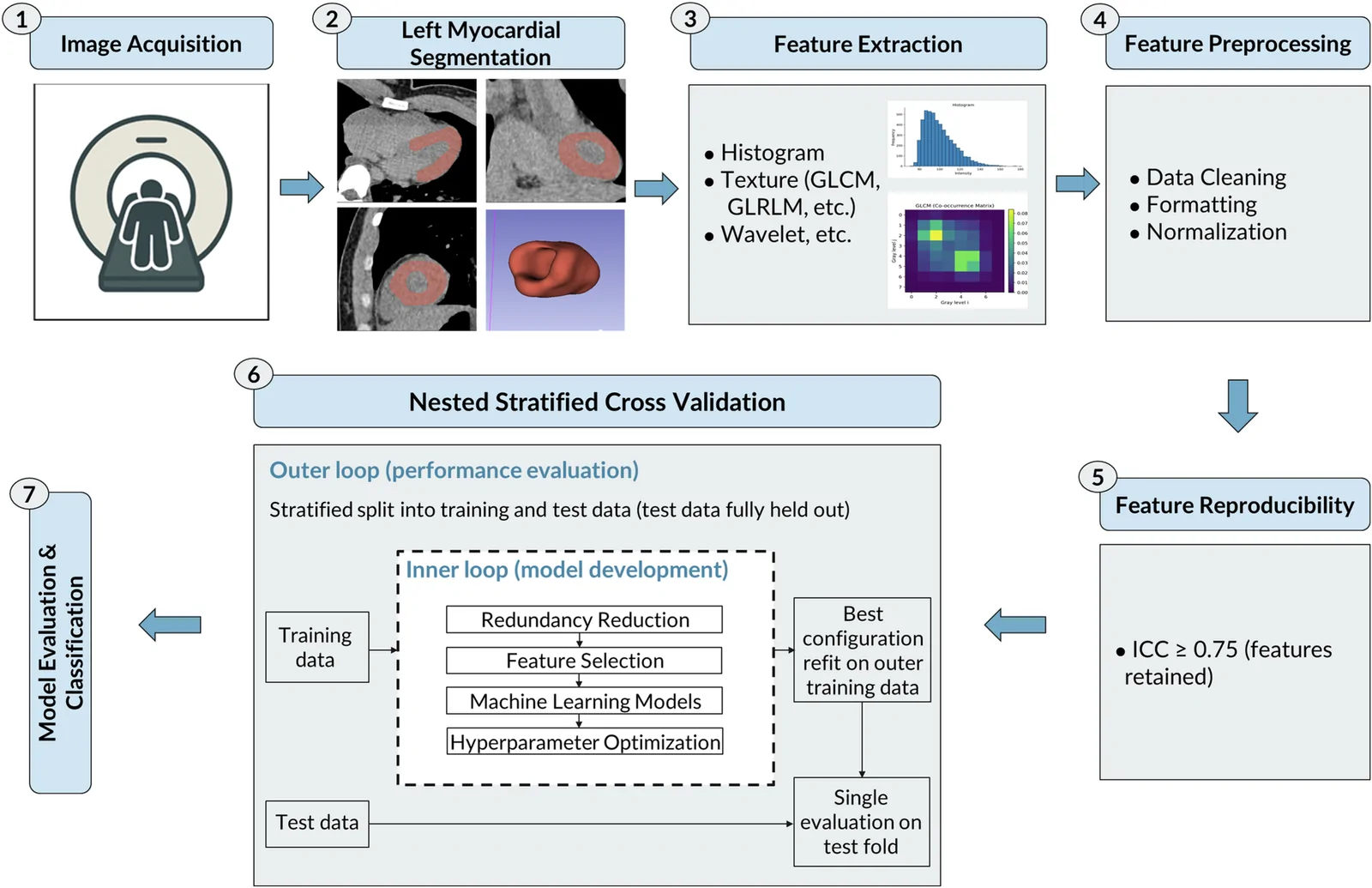
Flow diagram of data processing and machine learning. GLCM, Gray level co-occurrence matrix; GLRLM, Gray level run length matrix; ICC, Intraclass correlation coefficient

Segmentation of the entire left ventricular myocardium was performed independently by two readers in consensus (reader 1 with > 10 years of experience; reader 2 with > 2 years) using the freely available software tool *3D Slicer*. A semi-automatic approach was applied using the *Grow from Seeds* tool within the *Segment Editor* module, which employs an enhanced GrowCut algorithm. Seed regions were manually defined in axial, coronal, and sagittal planes and automatically expanded, followed by manual correction to exclude non-myocardial tissue. *Joint Smoothing* (smoothing factor 0.5) was applied to refine boundaries. The entire left ventricular myocardium was segmented as the volume of interest (Fig. 3). A notable feature of postmortem CT is a characteristic sedimentation phenomenon of blood within the vessels and cardiac chambers after death, allowing the myocardium to be relatively well distinguished from the ventricular cavity. In the absence of active circulation, blood redistributes, separating into a hypodense serum supernatant and a hyperdense layer of sedimented cellular components relative to the myocardium. These density differences allow for reliable delineation of the myocardium during the segmentation process. This postmortem effect can lead to increased density within the ventricular cavity despite the absence of contrast agent administration and accounts for the appearance observed in Fig. 3. In total, 212 volumes of interest were generated (106 cases × 2 readers; Fig. 3). Inter-reader consistency was assessed using a two-way mixed-effects intraclass correlation coefficient (ICC3) with absolute agreement [22].

### Radiomics feature extraction

Radiomic features were extracted from each left ventricular volume of interest using the *SlicerRadiomics* plugin (*PyRadiomics*) implemented in *3D Slicer* [23]. Images were resampled to an isotropic voxel size of 1 × 1 × 1 mm³ using *Linear* interpolation.

Previous studies have shown that using the full set of radiomic features, including those derived from filtered images (*e.g*., Laplacian-of-Gaussian (LoG) and wavelet transformations), does not reduce predictive performance [24–27]. A total of 1,037 features per case were extracted, comprising:

- 107 original features (18 first-order, 14 shape, and 75 texture features),
- LoG filtered features (σ = 3.0 mm and 5.0 mm), and
- Wavelet-transformed features.

The wavelet decomposition was performed along the three spatial axes using a combination of low-pass and high-pass filters.

All features were extracted in compliance with Image Biomarker Standardization Initiative–IBSI guidelines [28], given PyRadiomics’ broad alignment with this framework, apart from minor deviations in discretization and resampling. Further details are provided in the Supplementary materials.

### Feature normalization

A sign-based logarithmic transformation [29] was applied to normalize extreme values and preserve the sign of negative feature values. Further details are provided in the Supplementary materials.

### Radiomics feature reproducibility and redundancy reduction

Feature reproducibility was evaluated via inter-observer ICC using a two-way mixed-effects model (absolute agreement). ICC analysis is commonly used in radiomics to assess feature repeatability and reproducibility, particularly for evaluating the effect of inter-observer segmentation variability on radiomic features [30–32]. After exclusion of features with ICC < 0.75 [22], 586 features (≈ 50% of all extracted features) were retained as reproducible. This ICC-based analysis was applied as a predefined reproducibility assessment to identify and remove features sensitive to observer-dependent segmentation variability, rather than as an outcome-driven predictive feature-selection step. As ICC filtering is independent of the outcome labels, it does not introduce outcome-related information into the modeling process. By restricting the candidate feature set to features with acceptable inter-observer agreement, this step aimed to reduce segmentation-related variability and improve the robustness of the subsequent radiomics analysis.

To minimize multicollinearity, Spearman correlation analysis was performed within the inner loop of a nested cross-validation framework, as described in the following section. When ρ > 0.8, one of the correlated features was removed. Feature identifiers consisted of three components (*e.g.*, *original_glcm_Contrast*): image type, feature class, and feature name. Grouping by feature class provided the most efficient redundancy reduction strategy. Further details are provided in the Supplementary materials.

### Nested cross-validation and model development

Following feature reproducibility assessment (ICC filtering), model development and evaluation were performed using a nested stratified cross-validation framework to ensure unbiased performance estimation. Five outer folds and five inner folds were used, with stratification applied at both levels to preserve class balance.

The outer cross-validation loop was used exclusively for performance evaluation. In each outer fold, the dataset was divided into a training subset and a held-out test subset, with the test data remaining completely unseen during all data-dependent operations. Within each outer training subset, an inner cross-validation loop was used to perform correlation analysis, feature selection, and hyperparameter optimization using training data only.

The model configuration selected in the inner loop was refitted on the full outer training data and evaluated once on the corresponding outer test fold. This nested design ensured strict separation between training and test data and prevented information leakage during model development.

### Feature selection and dimensionality reduction

Feature selection was used to reduce dimensionality, limit overfitting, and enhance interpretability, and was performed exclusively on training data within the nested cross-validation framework. Three complementary strategies were implemented:

1. No additional selection (N.S.)—baseline; all features retained after reproducibility and correlation filtering were used without further ranking, serving as the reference feature set.
2. Embedded selection—random forest (RF) feature importance (mean decrease in impurity, MDI); feature importance was derived within the training data of each cross-validation fold using MDI, and the most important features were identified. Their selection frequency across outer folds was used to assess robustness.
3. Wrapper selection—recursive feature elimination using logistic regression* (RFE-LR);* features were iteratively removed within the training data of each cross-validation fold based on their contribution to the model. Features retained at the final step were considered most predictive, and their recurrence across outer folds was used to assess stability.

This strategy enabled comparative evaluation of model performance under varying feature sets.

### Machine learning and model evaluation

Supervised machine-learning models were implemented in Python using *scikit-learn* [33], *NumPy*, and related libraries. The objective was to classify cases as MI or control and identify predictive radiomic biomarkers. Evaluated classifiers included logistic regression (LR), support vector machine (SVM), k-nearest neighbors (kNN), naïve Bayes, random forest (RF), XGBoost, and CatBoost (Table 1).

**Table 1:** Machine-learning models and types

Model	Type
LR	Linear
SVM (SVC implementation)	Kernel-based
kNN	Instance-based
Naïve Bayes	Probabilistic
Random forest	Tree-based ensemble
XGBoost	Gradient boosting trees
CatBoost	Gradient boosting trees

*SVC* Support vector classifier

Model training, feature selection, and hyperparameter optimization were performed within the nested stratified cross-validation framework described above. Hyperparameters were optimized using grid search in the inner cross-validation loop, while model performance was evaluated exclusively on the held-out test data of the outer loop.

Model performance was assessed using the area under the receiver operating characteristic curve (AUROC), with additional reporting of accuracy, balanced accuracy, precision, sensitivity, specificity, and F1-score. Performance estimates were aggregated across outer folds and reported as mean values with corresponding standard deviations.

### Statistical analysis and software environment

All statistical analyses were performed in a Python-based environment (version 3.10.19). Radiomic feature extraction was conducted using *SlicerRadiomics* (git commit 8426cdf) and the *PyRadiomics* library (version 3.1.0) implemented in *3D Slicer* (version 5.8.1). Subsequent data processing, machine-learning modeling, and statistical evaluation were performed in Python using *NumPy*, *pandas* (version 2.3.3), *scikit-learn* (version 1.7.1), *matplotlib* (version 3.10.6), and *seaborn* (version 0.13.2) for data visualization.

Statistical testing and correlation analyses were conducted using *pingouin* (version 0.5.5). Tree-based and gradient-boosting classifiers were implemented using *XGBoost* (version 3.0.5) and *CatBoost* (version 1.2.7).

An overview of the workflow is illustrated in Fig. 3.

## Results

### Cohort characteristics and interrater analysis of segmentation

Demographic data, and infarction type are summarized in Table 2. The total number of ICC3 feature identifiers from both readers was 6221, comprising 308 excellent (≥ 0.90) and 278 good (≥ 0.75 and < 0.90) features, with a lowest 95% confidence interval of 0.65–0.82.

**Table 2:** Characteristics of the study cohort

	Cases with confirmed MI	Control cases
Number	55	51
Age		
Mean ± SD	73 ± 12	50 ± 14
Median	73	51
Range	43-92	21-80
Sex, number (%)		
Male	47	37
Female	8	14
Type of MI		
Acute/subacute MI	17	-
Reinfarction	38	-

*MI* Myocardial infarction, *SD* Standard deviation

### Feature selection

Two complementary feature selection approaches, RF and RFE-LR, were applied within the nested cross-validation framework. The 15 most stable radiomic features were identified based on their frequency of selection across outer folds. The complete lists of the 15 most stable features identified by each method are summarized in Table 3 (RF) and Table 4 (RFE-LR). Among the stable features, Informational Measure of Correlation 1 (Gray Level Co-occurrence Matrix [GLCM], Wavelet-LHH), 90th Percentile (First order, LoG σ = 5.0 mm), Strength (Neighborhood Gray-Tone Difference Matrix [NGTDM], LoG σ = 5.0 mm), and Correlation (Gray Level Co-occurrence Matrix [GLCM], Wavelet-HHH) were selected by both methods in at least two outer cross-validation folds.

**Table 3:** The 15 most stable features using random forest (RF)

Image type	Feature class	Feature name	No. of outer folds
Original	Shape	Least axis length	5
Original	GLDM	Dependence non-uniformity	4
LoG σ = 3.0 mm 3D	GLRLM	Gray level non-uniformity	5
LoG σ = 3.0 mm 3D	NGTDM	Coarseness	5
LoG σ = 3.0 mm 3D	GLDM	Gray level non-uniformity	5
LoG σ = 5.0 mm 3D	First order	90th percentile	5
LoG σ = 5.0 mm 3D	First order	Kurtosis	4
LoG σ = 5.0 mm 3D	NGTDM	Contrast	5
LoG σ = 5.0 mm 3D	NGTDM	Strength	5
Wavelet LHH	GLCM	Informational measure of correlation 1	5
Wavelet HHL	GLSZM	Size zone non-uniformity normalized	4
Wavelet HHL	GLDM	Dependence entropy	4
Wavelet HHH	GLCM	Maximum probability	5
Wavelet HHH	GLCM	Informational measure of correlation 1	5
Wavelet HHH	GLCM	Correlation	4

*3D* Three-dimensional, *GLCM* Gray level co-occurrence matrix, *GLDM* Gray level dependence matrix, *GLRLM* Gray level run length matrix, *GLSZM* Gray level size zone matrix, *H* High-pass filter, *L* Low-pass filter, *LoG* Laplacian-of-Gaussian, *NGTDM* Neighborhood gray tone difference matrix

**Table 4:** 15 most stable features using RFE-LR

Image type	Feature class	Feature name	No. of outer folds
Original	GLDM	Dependence entropy	3
Original	First order	Mean	2
LoG σ = 3.0 mm 3D	First order	Median	2
LoG σ = 3.0 mm 3D	GLRLM	Gray level non-uniformity normalized	3
LoG σ = 3.0 mm 3D	NGTDM	Busyness	2
LoG σ = 5.0 mm 3D	First order	90th percentile	4
LoG σ = 5.0 mm 3D	NGTDM	Strength	2
LoG σ = 5.0 mm 3D	GLDM	Dependence non-uniformity normalized	2
Wavelet LLL	NGTDM	Contrast	2
Wavelet LHH	GLCM	Informational measure of correlation 1	5
Wavelet LHH	GLCM	Contrast	2
Wavelet LHH	GLSZM	Gray level non-uniformity	2
Wavelet LHL	GLSZM	Zone entropy	2
Wavelet HLH	GLCM	Informational measure of correlation 1	2
Wavelet HHH	GLCM	Correlation	2

*3D* Three-dimensional, *GLCM* Gray level co-occurrence matrix, *GLDM* Gray level dependence matrix, *GLRLM* Gray level run length matrix, *GLSZM* Gray level size zone matrix, *H* High-pass filter, *L* Low-pass filter, *LoG* Laplacian-of-Gaussian, *NGTDM* Neighborhood gray tone difference matrix

### Model performance

**Fig. 4:**
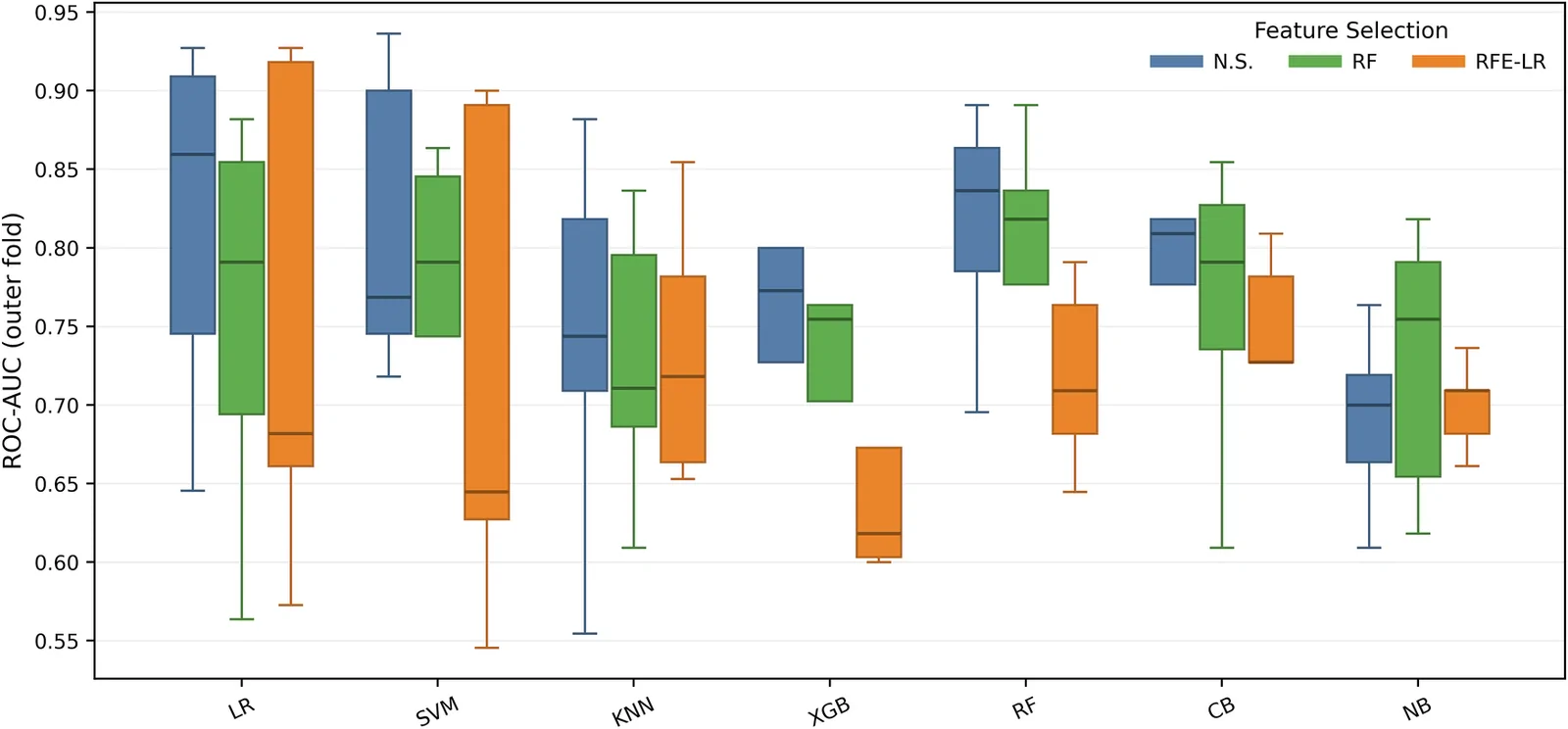
AUROC distributions across outer cross‑validation folds for all evaluated classifiers, with boxplots grouped by classifier and colored by feature‑selection strategy: N.S., random forest–based selection (RF), and RFE‑LR. Boxplots summarize the median, interquartile range, and range of AUROC values across folds. CB, CatBoost; kNN, k-nearest neighbors; LR, Logistic regression; NB, Naïve Bayes; RF, Random forest; SVM, Support vector machine; XGB, XGBoost

**Fig. 5:**
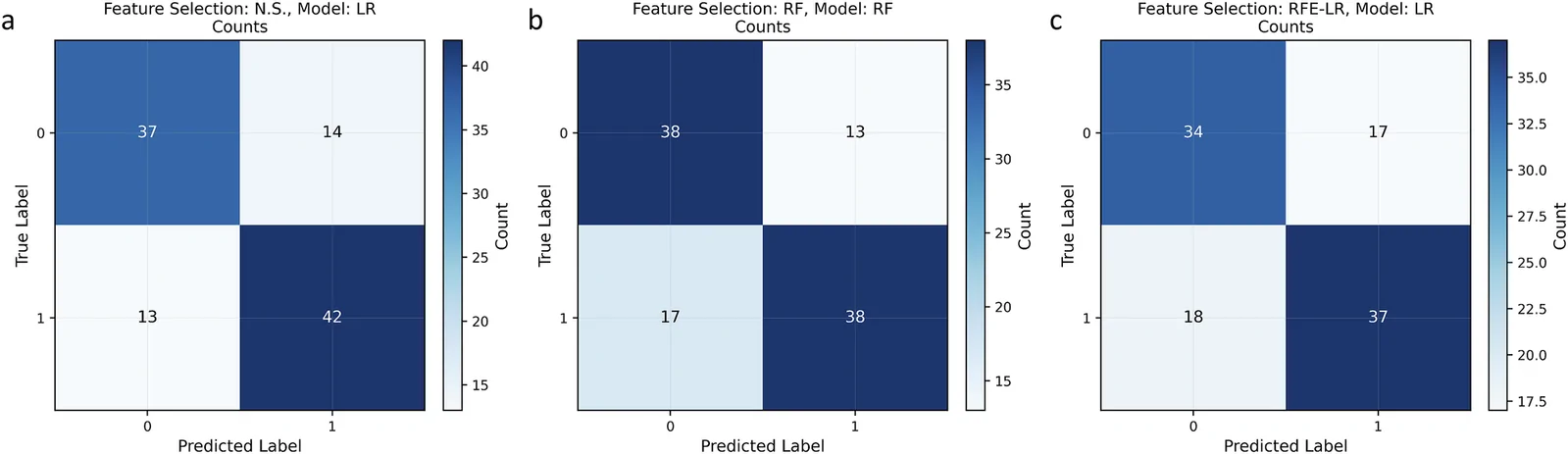
Confusion matrices for the top-performing classifier within each feature-selection strategy: **a** N.S. with LR classifier, **b** RF feature selection with RF classifier, and **c** RFE-LR feature selection with LR classifier. Predictions were pooled across outer cross-validation test folds. Class labels were assigned at a probability threshold of 0.5. LR, Logistic regression; N.S., No additional selection; RF, Random forest; RFE-LR, Recursive feature elimination using logistic regression

A graphical representation of the AUROC across all feature-selection strategies and classifiers is provided in Fig. 4, while Table 5 presents additional performance metrics in tabular form. Figure 5 further illustrates the confusion matrices derived for the top-performing classifier within each feature selection strategy.

**Table 5:** Performance of machine learning models

Models	Feature selection strategy	AUROC	Accuracy	Balanced accuracy	Precision	Sensitivity (recall, positive)	Specificity (recall, control)	F1-score (positive)
LR	N.S.	0.817 ± 0.12	0.746 ± 0.05	0.744 ± 0.05	0.76 ± 0.08	0.764 ± 0.14	0.724 ± 0.13	0.753 ± 0.07
	RF	0.757 ± 0.12	0.661 ± 0.12	0.658 ± 0.13	0.683 ± 0.14	0.691 ± 0.10	0.625 ± 0.22	0.681 ± 0.1
	RFE-LR	0.752 ± 0.16	0.671 ± 0.11	0.67 ± 0.11	0.717 ± 0.18	0.673 ± 0.08	0.667 ± 0.24	0.684 ± 0.09
SVM	N.S.	0.814 ± 0.1	0.745 ± 0.05	0.743 ± 0.06	0.755 ± 0.09	0.782 ± 0.1	0.704 ± 0.16	0.761 ± 0.05
	RF	0.751 ± 0.14	0.698 ± 0.09	0.696 ± 0.1	0.719 ± 0.12	0.727 ± 0.14	0.664 ± 0.21	0.713 ± 0.09
	RFE-LR	0.722 ± 0.16	0.651 ± 0.14	0.65 ± 0.14	0.679 ± 0.16	0.673 ± 0.14	0.627 ± 0.23	0.668 ± 0.12
kNN	N.S.	0.742 ± 0.12	0.707 ± 0.12	0.707 ± 0.13	0.752 ± 0.15	0.691 ± 0.08	0.724 ± 0.22	0.715 ± 0.1
	RF	0.728 ± 0.09	0.651 ± 0.09	0.651 ± 0.09	0.734 ± 0.11	0.636 ± 0.13	0.665 ± 0.12	0.651 ± 0.09
	RFE-LR	0.734 ± 0.08	0.699 ± 0.1	0.7 ± 0.1	0.699 ± 0.1	0.673 ± 0.22	0.727 ± 0.16	0.686 ± 0.13
XGBoost	N.S.	0.756 ± 0.13	0.679 ± 0.1	0.679 ± 0.11	0.699 ± 0.13	0.691 ± 0.08	0.667 ± 0.15	0.693 ± 0.09
	RF	0.742 ± 0.14	0.66 ± 0.1	0.66 ± 0.1	0.673 ± 0.1	0.673 ± 0.12	0.647 ± 0.11	0.671 ± 0.1
	RFE-LR	0.675 ± 0.12	0.68 ± 0.08	0.677 ± 0.08	0.67 ± 0.08	0.764 ± 0.1	0.591 ± 0.11	0.711 ± 0.08
Random forest	N.S.	0.814 ± 0.08	0.707 ± 0.04	0.708 ± 0.04	0.739 ± 0.08	0.691 ± 0.05	0.726 ± 0.11	0.711 ± 0.03
	RF	0.783 ± 0.12	0.717 ± 0.09	0.718 ± 0.09	0.749 ± 0.11	0.691 ± 0.08	0.745 ± 0.11	0.718 ± 0.09
	RFE-LR	0.718 ± 0.06	0.642 ± 0.05	0.641 ± 0.05	0.661 ± 0.08	0.655 ± 0.14	0.627 ± 0.15	0.65 ± 0.07
CatBoost	N.S.	0.718 ± 0.06	0.688 ± 0.06	0.689 ± 0.15	0.714 ± 0.15	0.655 ± 0.16	0.724 ± 0.13	0.683 ± 0.16
	RF	0.763 ± 0.1	0.67 ± 0.08	0.669 ± 0.08	0.689 ± 0.1	0.691 ± 0.05	0.647 ± 0.15	0.687 ± 0.06
	RFE-LR	0.72 ± 0.1	0.604 ± 0.05	0.604 ± 0.05	0.62 ± 0.06	0.636 ± 0.13	0.571 ± 0.14	0.621 ± 0.07
Naïve Bayes	N.S.	0.691 ± 0.06	0.565 ± 0.05	0.575 ± 0.04	0.674 ± 0.09	0.345 ± 0.1	0.804 ± 0.12	0.446 ± 0.08
	RF	0.727 ± 0.09	0.603 ± 0.06	0.609 ± 0.06	0.728 ± 0.12	0.436 ± 0.12	0.782 ± 0.22	0.528 ± 0.05
	RFE-LR	0.7 ± 0.03	0.557 ± 0.05	0.559 ± 0.04	0.591 ± 0.06	0.491 ± 0.22	0.627 ± 0.18	0.515 ± 0.13

Data are given as mean ± standard deviation

*kNN* k-nearest neighbors, *N.S*. No additional selection, *RF* Random forest, *RFE-LR* Recursive feature elimination using logistic regression, *SVM* Support vector machine

Overall, LR with N.S. achieved the highest and most consistent predictive performance, with an AUROC of 0.817 ± 0.12 (mean ± standard deviation). This configuration yielded an accuracy of 0.746 ± 0.05, a sensitivity of 0.764 ± 0.14, and a specificity of 0.724 ± 0.13 (mean ± standard deviation).

A comparable performance profile was observed for SVM and RF with N.S., with mean AUROC values of 0.814 ± 0.10 and 0.814 ± 0.08, with corresponding accuracies of 0.745 ± 0.05 and 0.707 ± 0.04, sensitivities of 0.782 ± 0.10 and 0.691 ± 0.05, and specificities of 0.704 ± 0.16 and 0.726 ± 0.11, respectively (mean ± standard deviation).

For most of the remaining classifiers, mean AUROC values were generally below 0.8 and ranged between 0.7 ± 0.03 and 0.783 ± 0.12, regardless of the applied feature selection strategy. Only NB with N.S. and XGBoost with RFE-LR showed mean AUROC values below 0.7 (0.691 ± 0.06 and 0.675 ± 0.12, respectively; mean ± standard deviation).

When comparing feature selection strategies, N.S. achieved predominantly higher AUROC values than RF-based and RFE-LR feature selection across most classifiers, including LR, SVM, kNN, and XGBoost, and RF. By contrast, for CatBoost and NB AUROC values were lower with N.S. than with RF-based and RFE-LR feature selection strategies.

## Discussion

This study demonstrates that radiomics-based machine-learning analysis of noncontrast PMCT enables overall good performance in differentiating between cases with autopsy-confirmed MI and controls without cardiac pathology, even in the absence of blood flow and contrast enhancement. To the best of our knowledge, the present study represents the first systematic application of radiomics to postmortem imaging for MI determination, establishing a foundation for the development of quantitative imaging biomarkers in cardiopathology. Initial radiomics-oriented PMCT studies have addressed other forensic questions, such as estimating the time since death [34, 35].

Among all evaluated machine-learning models, LR (N.S.) showed the highest discriminative performance (AUROC: 0.817 ± 0.12, mean ± standard deviation). For comparison, previous *in vivo* texture analysis and radiomics studies investigating MI in cardiac CT (both with and without contrast enhancement) have reported comparable results with slightly lower or higher values. Mannil et al [13] demonstrated that noncontrast, low-dose cardiac CT can detect acute and chronic MI invisible to the human eye using texture analysis and machine-learning approaches, achieving an AUROC of 0.78 (95% confidence interval not available). While, O’Brien et al [14] further showed that delayed contrast-enhanced cardiac CT, *by applying radiomics and machine-learning techniques*, allows accurate detection of myocardial scar tissue, reaching an AUROC of 0.88 (95% confidence interval 0.87–0.90). These differences may, at least in part, reflect variations in image acquisition, the use of contrast enhancement, and overall study design, and suggest that the present contrast-free PMCT approach offers competitive performance despite its inherent limitations. Although MRI-based techniques are less directly comparable to our CT-based postmortem approach, texture analysis/radiomics studies using cardiac MRI have likewise demonstrated the ability to detect or characterize MI, reporting higher AUROC values ranging from 0.860 ± 0.060 to 0.930 ± 0.030 [15–18]. The higher performance observed in MRI-based studies may be attributable to its superior soft-tissue contrast and the absence of postmortem-related imaging artifacts. Nevertheless, these findings support the potential of radiomics-based approaches to provide meaningful diagnostic information across different imaging modalities, including contrast-free postmortem CT.

In contrast to these *in vivo* studies, our noncontrast PMCT approach eliminates confounding factors such as cardiac motion, tissue perfusion, and contrast enhancement. The good classification performance achieved under such static conditions demonstrates that intrinsic attenuation heterogeneity alone captures the structural disorganization characteristic of infarcted myocardium. This supports the concept that the radiomic information reflects genuine tissue-level alterations, such as necrosis, edema, and fibrosis, rather than perfusion-related artifacts.

In this context, our results showed that a well-defined and reproducible subset of radiomic features provided robust separation between infarcted and non-infarcted myocardium on noncontrast PMCT. Notably, LR and SVM achieved diagnostic performance comparable to or exceeding that of more complex ensemble models (*e.g*., gradient boosting), suggesting that increased model complexity does not necessarily translate into improved performance.

The most relevant image features comprised both first-order intensity measures and higher-order textural descriptors, reflecting changes in myocardial density and structural organization. Four radiomic features were consistently identified by both feature selection approaches (RF and RFE-LR) when accounting for stability across outer cross-validation folds. Two originated from filtered images at a coarse spatial scale: 90th percentile (first order, LoG σ = 5 mm), highlighting high-intensity regions, and Strength (Neighborhood Gray-Tone Difference Matrix [NGTDM], LoG σ = 5 mm), characterizing the magnitude of local gray-level differences. Two additional features were derived from wavelet-transformed images: Informational Measure of Correlation 1 (Gray Level Co-occurrence Matrix [GLCM], Wavelet-LHH) and Correlation (Gray Level Co-occurrence Matrix [GLCM], Wavelet-HHH), capturing voxel-to-voxel gray-level dependencies and local textural organization at finer, directionally sensitive scales [28]. Together, these complementary features describe how infarction alters myocardial structure across multiple spatial levels—from global density reduction to fine-scale loss of textural coherence.

The use of LoG- and wavelet-transformed images enabled texture characterization across multiple spatial scales. Original features described intensity and texture patterns present in the unfiltered myocardium. LoG filtering isolated scale-specific structural variations—fine details at small σ and broader textural patterns at large σ—by highlighting regions of rapid intensity change. Wavelet decomposition further separated texture into directional and frequency-specific components, enabling detection of both coarse and fine structural irregularities. Combining these complementary image domains provided a more comprehensive assessment of myocardial injury and likely contributed to the good diagnostic performance observed [24–27]. The consistent selection of these multi-scale, reproducible features across independent algorithms suggests the presence of a stable radiomic profile of MI.

From a forensic and pathological perspective, PMCT radiomics represents a promising non-invasive approach within PMCT-based cause-of-death diagnostics. Quantitative texture features may support diagnosis of MI, particularly in cases where autopsy is limited due to resource constraints [4, 5] or refused due to cultural, religious, and personal considerations [6–8]. In these settings, where autopsy is constrained or not performed, PMCT may represent the primary or sole source of diagnostic information. Radiomics-based MI detection is therefore intended to further enhance the diagnostic value of PMCT data. As high-quality postmortem imaging becomes increasingly available, such approaches could help standardize and objectify cause-of-death assessment. In this context, emerging CT technologies, such as spectral imaging including dual-energy and photon-counting CT, may further enhance myocardial tissue characterization.

From a clinical standpoint, our findings suggest the broader potential of contrast-free radiomic-based machine learning analysis for cardiac imaging. However, direct correlation between the present postmortem results and *in vivo* noncontrast CT cannot be assumed and requires further validation. Whether similar approaches could support the assessment of MI *in vivo* remains to be investigated, particularly with regard to their potential application in patients with contraindications to contrast agents.

This exploratory, single-center study has several limitations. The retrospective design and limited cohort size restrict generalizability, and scanner-specific acquisition parameters may influence feature stability. External validation on larger, multi-center datasets is required to confirm robustness across scanners and postmortem intervals. Harmonized reconstruction protocols and systematic evaluation of postmortem interval effects would further enhance reproducibility.

While our analysis focused on binary MI detection, future studies should investigate differentiation of acute/subacute, and later MI stages, as previously shown feasible in MRI [15, 16]. Integration of co-registered histopathology could strengthen biological interpretability, and deep-learning architectures trained on radiomic features may enable automatic detection and spatial localization of infarcted regions.

In conclusion, our study demonstrates that noncontrast PMCT combined with radiomics-based machine-learning analysis can detect MI with good diagnostic performance. These findings offer a basis for reliably assessing MI with PMCT in forensic and pathological settings and suggest a potential translational relevance of contrast-free CT techniques for improving MI detection in clinical settings, although their applicability to *in vivo* imaging requires further investigation.

## Supplementary information

**Additional file 1: Table S1** Feature distribution by image type. Shape features were extracted only from Original image type. **Table S2** Complete set of unique radiomic features extracted across all image types. **Table S3** Feature grouping and feature count. **Figure S1** Sign-Based Log Transformation. **Figure S2** Correlation maps of the GLCM features a) before and b) after the spearman analysis. **Figure S3** Feature stability across outer cross-validation folds.

## Data Availability

The datasets used and/or analyzed during the current study are available from the corresponding author on reasonable request.

## Abbreviations

ICC: Intraclass correlation coefficient
kNN: k-nearest neighbors
LoG: Laplacian-of-Gaussian
LR: Logistic regression
MI: Myocardial infarction
MRI: Magnetic resonance imaging
N.S.: No additional selection
PMCT: Postmortem computed tomography
RF: Random forest
RFE-LR: Recursive feature elimination using logistic regression
SVM: Support vector machine
