# Supplementary material for: Detection of myocardial infarction by postmortem CT combined with radiomics-based machine-learning analysis
Source: Eur Radiol Exp. 2026 Sep 11;10:130. doi: 10.1186/s41747-026-00800-4 (PMC13569735; doi:10.1186/s41747-026-00800-4)
Supplement: Supplementary file 1 [file 41747_2026_800_MOESM1_ESM.pdf]

Detection of myocardial infarction by postmortem CT combined with radiomics-based machine-learning analysis

ELECTRONIC SUPPLEMENTARY MATERIAL

Supplementary materials

Additional details regarding the Methods are provided below to ensure transparency and reproducibility of the study.

Feature preprocessing and normalization

Following radiomic feature extraction, non-informative metadata entries labeled as diagnostics (e.g., hash values and software version information) were excluded. After this step, 1,037 radiomic features per patient were retained for preprocessing. These features comprised shape, first-order, and texture feature classes. A detailed breakdown and complete list of extracted features are provided in Table S1.
Radiomic features varied in scale; therefore, a sign-based logarithmic transformation [1] was applied to all features, defined as:

$$f(x) = sign(x) * log(1 + |x|)$$

Where,

$$sign(x) = \begin{cases} 1, & if x > 0 \\ -1, & if x < 0 \\ 0, & if x = 0 \end{cases}$$

This logarithmic normalization compressed the extreme values and ensured not just the positive but the negative values are also appropriately retained. Figure S1 illustrates the behavior of the sign-based log transformation for both the positive and negative values. The complete set of unique radiomic features extracted across all image types is provided in Table S2.

Radiomics feature extraction

A bin width of 25 was used for grey-level discretisation. To prevent negative voxel intensities from being squared during processing, voxelArrayShift = +1024 was applied, and data were converted from *char* to *float* using *CastImageFilter*.

LoG- and wavelet-derived features were limited to first order and texture feature classes.

The LoG filter enhances edges and highlights local intensity variations by convolving the image with a Gaussian kernel followed by the Laplacian operator, thereby emphasizing fine textural structures at different spatial scales.

Wavelet decomposition along the three spatial axes using combinations of low-pass (L) and high-pass (H) filters yielded eight image types (LLL, LLH, LHL, LHH, HLL, HLH, HHL, and HHH), each capturing different spatial frequency patterns. Low-pass filters preserve coarse structures, whereas high-pass filters enhance fine details and edges, enabling extraction of complementary radiomic features from multiple frequency domains.

Radiomics feature reproducibility and redundancy reduction

Radiomic feature extraction is known to be susceptible to variability arising from differences in image acquisition, segmentation, and feature computation. In particular, inter-observer variability in manual or semi-automatic segmentations can substantially affect feature stability. To address this, inter-observer reproducibility was assessed in accordance with previous recommendations for radiomics studies [2].

Feature reproducibility was quantified using the intraclass correlation coefficient (ICC) based on a two-way mixed-effects model with absolute agreement, following the reliability framework described by Koo and Li (2016). According to this framework, ICC values below 0.50 indicate poor reliability, values between 0.50 and 0.75 moderate reliability, values between 0.75 and 0.90 good reliability, and values above 0.90 excellent reliability. In the present study, features with ICC $\geq$ 0.75 were considered reproducible and retained for further analysis, whereas features below this threshold were excluded as potentially unstable.

To inform the design of the redundancy reduction strategy used in the final modeling pipeline, an exploratory correlation analysis was performed as a pre-study outside the cross-validation framework. Redundancy among ICC-filtered features was assessed using Spearman correlation (ρ > 0.8) under three grouping strategies: (i) grouping by image type and feature class, (ii) grouping by feature class, and (iii) grouping by image type. Radiomic feature identifiers consisted of image type, feature class, and feature name (e.g., original_glcm_Contrast). Within each strategy, one feature from each highly correlated pair was removed.

The number of removed and retained features under each grouping strategy is summarized in Table S3. Grouping by feature class provided the most effective redundancy reduction while preserving feature diversity across image types and was therefore selected for use in the

nested cross-validation–based pipeline, where correlation filtering was applied within the inner training folds. Figure S2 illustrates the correlation maps of GLCM features before and after Spearman correlation analysis.

Feature stability across outer cross-validation folds

Feature stability was assessed across the five outer folds of the nested cross-validation framework. Stability was defined as the number of folds (maximum = 5) in which a radiomic feature was selected during model development. Figure S3 shows the selection frequency of radiomic features across the outer folds.

Tables

Table S1 Feature distribution by image type. Shape features were extracted only from Original image type

Image type	Shape	First-order	Texture	Total
Original	14	18	75	**107**
LoG (σ = 3.0 mm)	-	18	75	**93**
LoG (σ = 5.0 mm)	-	18	75	**93**
Wavelet	-	144	600	**744**
**Total**	**14**	**198**	**825**	**1037**

Table S2 Complete set of unique radiomic features extracted across all image types

Feature Class	Feature Name
firstorder	10Percentile
	90Percentile
	Energy
	Entropy
	InterquartileRange
	Kurtosis
	Maximum
	Mean
	MeanAbsoluteDeviation
	Median
	Minimum
	Range
	RobustMeanAbsoluteDeviation
	RootMeanSquared
	Skewness
	TotalEnergy
	Uniformity
	Variance
glcm	Autocorrelation
	ClusterProminence
	ClusterShade
	ClusterTendency
	Contrast
	Correlation
	DifferenceAverage
	DifferenceEntropy
	DifferenceVariance
	Id
	Idm
	Idmn
	Idn
	Imc1
	Imc2
	InverseVariance
	JointAverage
	JointEnergy
	JointEntropy
	MCC
	MaximumProbability
	SumAverage
	SumEntropy
	SumSquares
gldm	DependenceEntropy
	DependenceNonUniformity
	DependenceNonUniformityNormalized
	DependenceVariance
	GrayLevelNonUniformity
	GrayLevelVariance
	HighGrayLevelEmphasis
	LargeDependenceEmphasis
	LargeDependenceHighGrayLevelEmphasis

	LargeDependenceLowGrayLevelEmphasis
	LowGrayLevelEmphasis
	SmallDependenceEmphasis
	SmallDependenceHighGrayLevelEmphasis
	SmallDependenceLowGrayLevelEmphasis
glrlm	GrayLevelNonUniformity
	GrayLevelNonUniformityNormalized
	GrayLevelVariance
	HighGrayLevelRunEmphasis
	LongRunEmphasis
	LongRunHighGrayLevelEmphasis
	LongRunLowGrayLevelEmphasis
	LowGrayLevelRunEmphasis
	RunEntropy
	RunLengthNonUniformity
	RunLengthNonUniformityNormalized
	RunPercentage
	RunVariance
	ShortRunEmphasis
	ShortRunHighGrayLevelEmphasis
	ShortRunLowGrayLevelEmphasis
glszm	GrayLevelNonUniformity
	GrayLevelNonUniformityNormalized
	GrayLevelVariance
	HighGrayLevelZoneEmphasis
	LargeAreaEmphasis
	LargeAreaHighGrayLevelEmphasis
	LargeAreaLowGrayLevelEmphasis
	LowGrayLevelZoneEmphasis
	SizeZoneNonUniformity
	SizeZoneNonUniformityNormalized
	SmallAreaEmphasis
	SmallAreaHighGrayLevelEmphasis
	SmallAreaLowGrayLevelEmphasis
	ZoneEntropy
	ZonePercentage
	ZoneVariance
ngtdm	Busyness
	Coarseness
	Complexity
	Contrast
	Strength
shape	Elongation
	Flatness
	LeastAxisLength
	MajorAxisLength
	Maximum2DDiameterColumn
	Maximum2DDiameterRow
	Maximum2DDiameterSlice
	Maximum3DDiameter
	MeshVolume
	MinorAxisLength
	Sphericity
	SurfaceArea

	SurfaceVolumeRatio
	VoxelVolume

Table S3 Feature grouping and feature count

**Group By**	**Features Compared (E.g.)**	**Removed Features Count**	**Remaining Feature Count**
Image type and feature class	**original_glcm**_Contrast, **original_glcm**_Energy	437	149
Feature class	original_**glcm**_Contrast, wavelet-HLL_**glcm**_Energy	524	**62**
Image type	**wavelet-HLL**_firstorder_Mean, **wavelet-HLL**_glcm_Contrast	522	64

Figures

Figure S1 Sign-Based Log Transformation

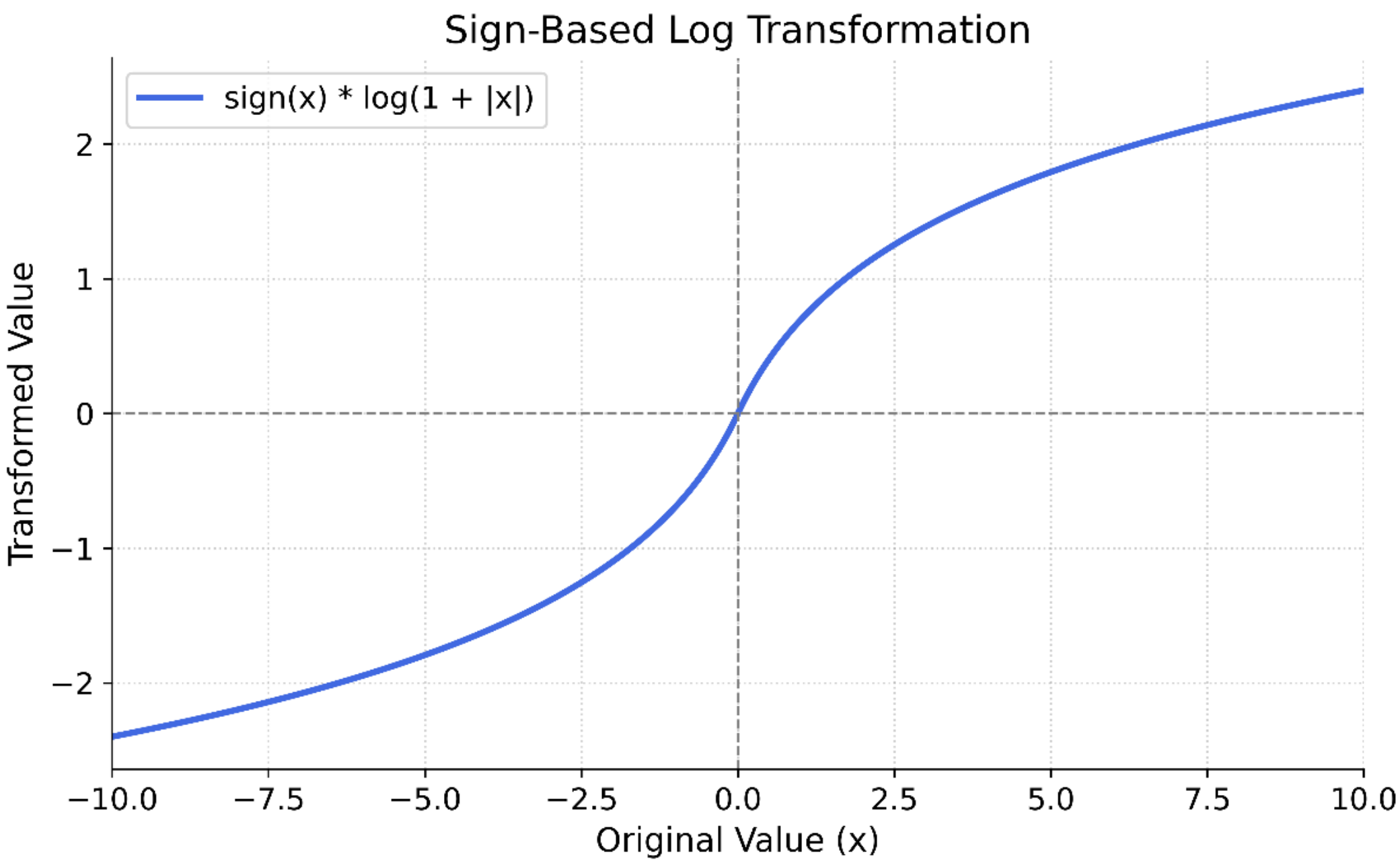

Figure S2 Correlation maps of the GLCM features a) before and b) after the spearman analysis

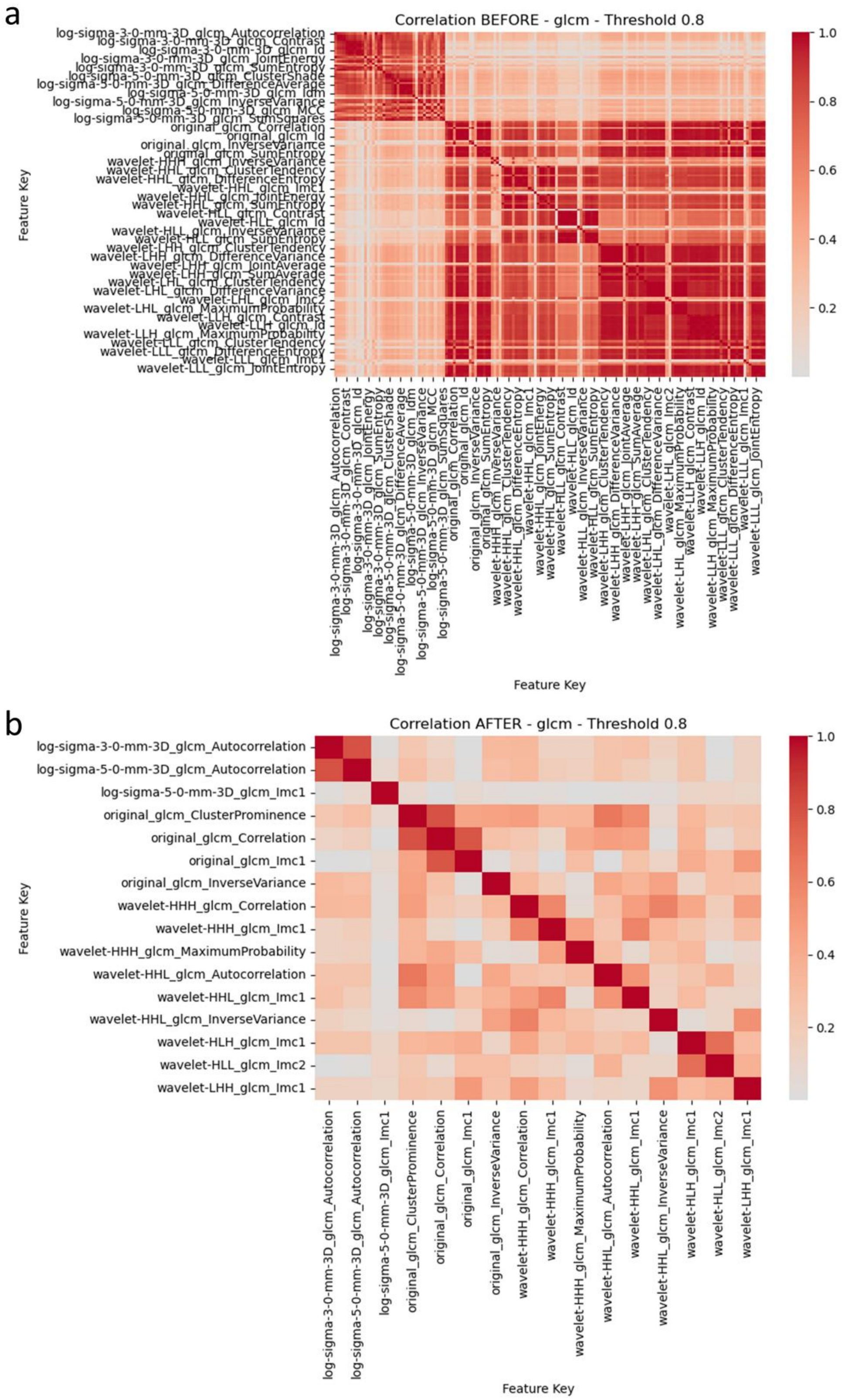

Figure S3 Feature stability across outer cross-validation folds

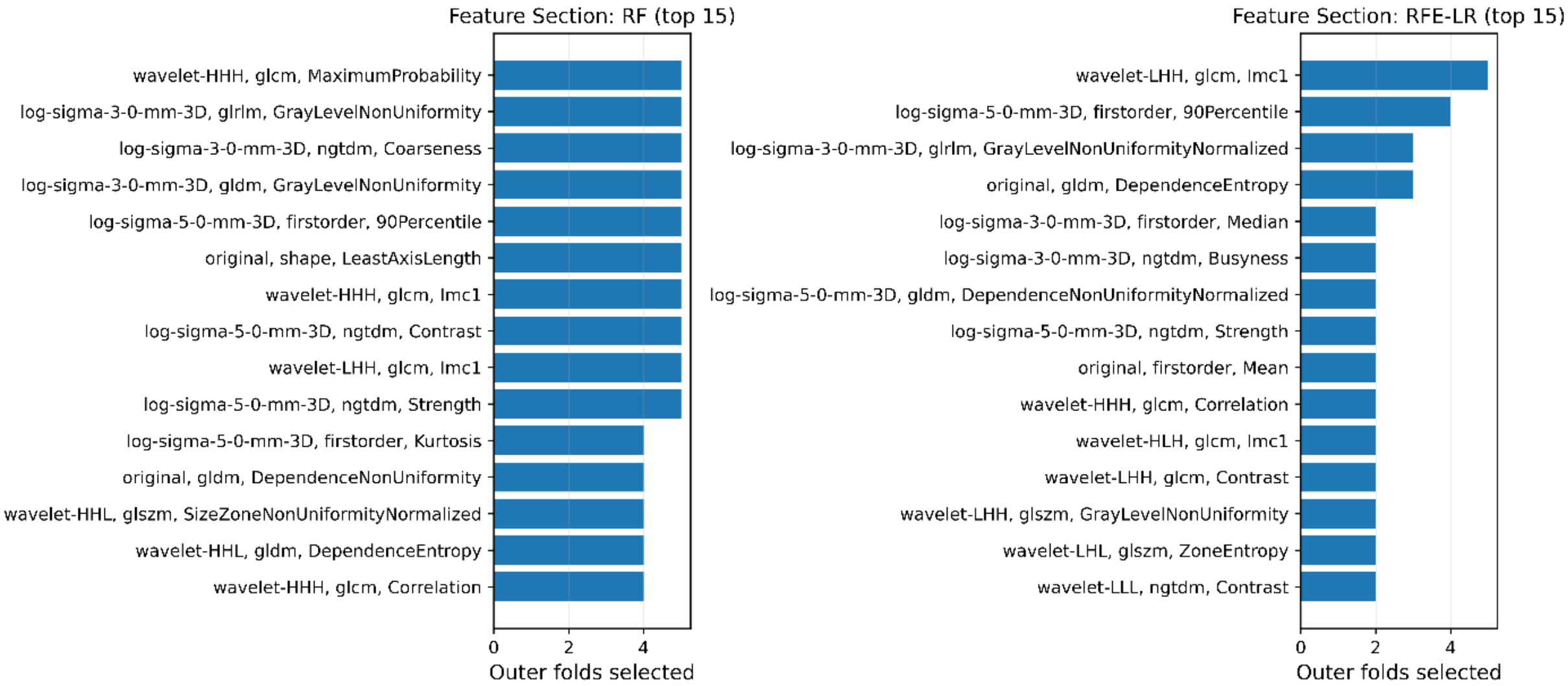

References

1. John JA, Draper NR (1980) An Alternative Family of Transformations. Applied Statistics 29(2):190.
2. van Timmeren JE, Cester D, Tanadini-Lang S, Alkadhi H, Baessler B (2020) Radiomics in medical imaging-“how-to” guide and critical reflection. Insights Imaging 11(1):91.
